# Two-stage meta-analysis of survival data from individual participants using percentile ratios

**DOI:** 10.1002/sim.5516

**Published:** 2012-07-24

**Authors:** Jessica K Barrett, Vern T Farewell, Fotios Siannis, Jayne Tierney, Julian P T Higgins

**Affiliations:** a MRC Biostatistics UnitCambridge, U.K; b Department of Mathematics, University of AthensAthens, Greece; c MRC Clinical Trials UnitLondon, U.K

**Keywords:** individual patient data, meta-analysis, survival data

## Abstract

Methods for individual participant data meta-analysis of survival outcomes commonly focus on the hazard ratio as a measure of treatment effect. Recently, Siannis *et al.* (2010, *Statistics in Medicine* 29:3030–3045) proposed the use of percentile ratios as an alternative to hazard ratios. We describe a novel two-stage method for the meta-analysis of percentile ratios that avoids distributional assumptions at the study level. Copyright © 2012 John Wiley & Sons, Ltd.

## 1. Introduction

In a review of individual participant data (IPD) meta-analyses published during the years 1999 to 2001, Simmonds *et al.* 1 found that in practice meta-analysis of IPD was most frequently conducted using simple two-stage methods. For example, in the meta-analysis of survival IPD, hazard ratios can be estimated for each study individually in the first stage using a proportional hazards model, or approximated by a log-rank statistic (for example, 2). Individual study results can then be combined using standard random-effects or fixed-effects meta-analysis in the second stage.

In the analysis of a single survival study, the hazard ratio is a commonly used measure of treatment effect and is therefore a natural quantity to consider when undertaking a meta-analysis. However, an assumption of proportional hazards is even more restrictive in meta-analysis because it is imposed on multiple studies. If the proportional hazards assumption does not hold for some studies, then the estimated hazard ratio depends on the length of follow-up in those studies, and meta-analysis may not be appropriate. Methods that relax the proportional hazards assumption, the majority of which focus on a measure or measures of treatment effect that are based on the hazard ratio, have been proposed. For example, Moodie *et al.* 3 meta-analysed a function that can be interpreted as a log-transformed weighted average of hazard ratios over time for each trial. Arends *et al.* 4 extended the model of Dear *et al.* 5 by modelling the survival probabilities in treatment and control groups using a multivariate, mixed-effects model. The treatment effect in the model of Arends *et al.* is the hazard ratio, which can be time varying when treatment-by-time interaction terms are included in the model. Fiocco *et al.* 6 described a piecewise-constant hazard model, where hazards for the treatment and control arms are modelled using a bivariate frailty model. And more recently, Ouwens *et al.* 7 have proposed a meta-analysis model in which hazard ratios are time varying and expressed in terms of the shape and the scale parameters of parametric survival curves. The methods we propose here provide an alternative approach to the use of time-varying hazard ratios in this context.

In a recent paper, Siannis *et al.* 8 considered the percentile ratio as an alternative measure of treatment effect in survival IPD meta-analysis when proportional hazards assumptions are not appropriate. The percentile ratio *q*_*k*_ at percentile level *k* comparing the survival distributions of two groups is defined as


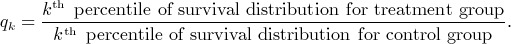
(1)

The median ratio at *k* = 0.5 represents the expected ratio of times at which half of the population will fail in the treatment group compared with the control group. Similarly, for other percentile levels *k*, the percentile ratio is the expected ratio for the time at which 100*k%* of the population will fail in the treatment group compared with the control group. Because the interpretation of percentile ratios can be so easily explained, their use could lead to clearer, more practical understanding of survival differences between treatment groups. In general, percentile ratios may vary across percentile levels. If the survival distributions in question are accelerated failure time models, however, the percentile ratio *q* is constant across all values of *k* 8.

Siannis *et al.* estimated percentile ratios using a one-stage parametric model with data at the individual study level being modelled using either accelerated failure time or proportional hazards distributions. In the simplest version of the model, accelerated failure time distributions were used to model the data at the study level. In this case, the combined percentile ratio *q* is constant across percentile levels and can be modelled using either fixed or random effects. The proposed framework is very general, however, and could be used to model any choice of distribution at the study level. This was illustrated using a combination of accelerated failure time and proportional hazards models.

Motivated by the popularity of simple two-stage analyses, we propose an alternative, two-stage method for meta-analysis of percentile ratios, which in addition avoids all distributional assumptions in the first stage. In stage 1, we use Kaplan–Meier estimates of the survivor functions for the treatment and control groups to estimate percentile ratios and their variance–covariance matrix. In stage 2, percentile ratios are combined using either univariate or multivariate, random-effects meta-analysis (see 9 for an overview of multivariate meta-analysis). The pros and cons of using multivariate meta-analysis in this context are explored in the analysis of an example data set.

The layout of the paper is as follows. In Section 2, we describe the new two-stage method and explore its properties using a simulation study in Section 3. We apply the method to an example data set in Section 4 and conclude with a discussion in Section 5.

## 2. Two-stage meta-analysis of percentile ratios

In this section, we describe our methods in more detail. In Section 2.1, we describe stage 1 of the analysis, in which a vector of log percentile ratios (logPRs) and its variance–covariance matrix is estimated for each study. In Section 2.2, we describe how estimated logPRs from several studies can be combined using multivariate meta-analysis.

### 2.1 Stage 1: estimation of log percentile ratios

We focus here on the analysis of the data from a single study. The aim is to estimate a vector of logPRs **q**_*i*_ = (*q*_*i*1_, …, *q*_*iK*_)^T^ for each study *i*, where *q*_*ik*_ denotes the percentile ratio in study *i* at percentile level *k*, and its variance–covariance matrix **S**_*i*_. Methods for combining these estimates will be discussed in Section 2.2. To estimate *q*_*ik*_, we first estimate the *k*th percentile in the treatment and control groups for study *i*, which we denote 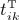 and 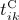, respectively, and use


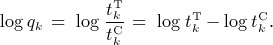
(2)

To simplify the notation, we will ignore study index *i* for the remainder of this section. We will assume throughout that censoring is non-informative, that is, it occurs independently of survival.

The percentile *t*_*k*_ at percentile level *k* of a distribution of survival times is defined by the equation *S*(*t*_*k*_) = *k*, where *S*() is the survivor function. We estimate *t*_*k*_ from a Kaplan–Meier estimate of the survivor function 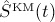 as follows


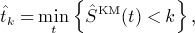
(3)

where the minimisation in (3) is necessary because of the steplike nature of the estimated survival curve 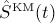. In a graph of 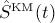 against *t*, this corresponds to the value of *t* for which a horizontal line at *S*(*t*) = *k* crosses the estimated survival curve. If there is an interval *t*_1_ *≤ t* < *t*_2_ for which *S*(*t*) = *k* exactly, then the percentile is estimated to be the midpoint of that interval:


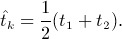
(4)

Suppose 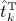 and 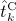 are the estimated *k*th percentiles in the treatment and control groups, respectively. Then, an estimate for the logPR is given by substituting the estimates 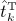 and 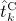 in Equation (2).

Sander 10 demonstrated asymptotic normality for quantile estimates of survival distributions derived from Kaplan–Meier curves under certain conditions and further discussed by Roth 11 and Reid 12 (we thank a referee for pointing out these references). However, estimation of the variance–covariance matrix **S** for quantile estimates is not straightforward. We describe two methods, the first of which is based on asymptotic approximations and is relatively fast to compute. The second method uses bootstrapping but is computationally more intensive.

#### 2.1.1 Asymptotic variance estimation

A simple asymptotic expression for the variance of an estimated log-percentile 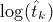 is given by


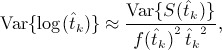
(5)

where *f*(*t*) is the probability density function of the survival distribution (see Appendix A.1 for a derivation). To estimate 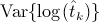, we use Equation (5) with 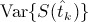 and *f*(*t*) replaced by suitable estimates. 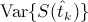 is taken to be 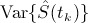 (theorem 2 of 11), which is estimated by Greenwood's formula. The estimation of *f*(*t*) is less straightforward but can be carried out using the presmooth package in R 13. A brief explanation of presmoothed density estimation is given in Appendix B.

Having estimated the variances of both treatment and control group log percentiles, an estimate of the variance of the estimated logPR is given by


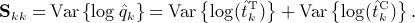
(6)

An asymptotic expression to estimate the covariance between two estimated log percentiles 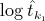 and 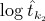 is given by


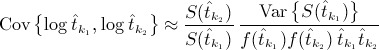
(7)

(see Appendix A.2 for a derivation). When *k*_1_ = *k*_2_, Equation (7) reduces to the variance formula of Equation (5). Var{*S*(*t*)}and *f*(*t*) can again be estimated using Greenwood's formula and the presmooth package, respectively. The estimated covariance matrix **S** between two logPRs is then given by


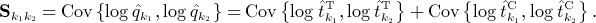
(8)

#### 2.1.2 Bootstrap variance estimation

Variance–covariance matrices could alternatively be estimated by bootstrapping. The validity of bootstrap methods for the case of censored data has been demonstrated by Efron 14 (see also 15 for a summary of bootstrap methods). For a study with *n*_T_ and *n*_C_ participants in the treatment and control groups, respectively, a bootstrap sample is constructed by making *n*_T_ random draws with replacement from the treatment group and *n*_C_ from the control group. A large number *B* of bootstrap samples are drawn, and each is used to estimate a vector of logPRs 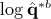 for *b* = 1, …, *B*. Then, an estimate of the variance–covariance matrix **S** of 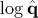 is given by


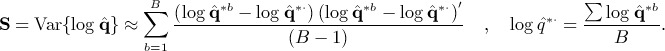
(9)

### 2.2 Stage 2: meta-analysis

The output from stage 1 of the analysis is an estimated logPR or vector of logPRs from each study, 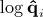, along with an estimated variance or variance–covariance matrix **S**_*i*_. **S**_*i*_ can be estimated using Equation (8) or (9) but, for the purposes of the meta-analysis, is assumed fixed and known. When a single percentile level *k* is of interest, logPRs can be combined using standard random-effects meta-analysis as follows. The estimated logPR from study *i*, 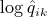, is assumed to be normally distributed about the true logPR in study *i*, log *q*_*ik*_, with variance **S**_*i*,*kk*_:


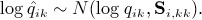
(10)

The true logPR in study *i*, log *q*_*ik*_, is then assumed to be normally distributed about the combined logPR *q*_*k*_ with between-studies variance 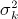:


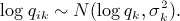
(11)

If a vector of logPRs from each study are to be combined, this can be performed either by using a separate univariate meta-analysis at each percentile level or by combining logPRs at all levels simultaneously using multivariate meta-analysis. It has been argued that multivariate meta-analysis is preferable when combining multiple correlated outcomes because estimates of combined effect sizes borrow strength across outcomes through the correlations between them 16. For a multivariate meta-analysis, we assume that 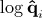 has a multivariate normal distribution


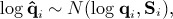
(12)

where log**q**_*i*_ is a vector of true, underlying logPRs in study *i*. **S**_*i*_ is the within-study variance–covariance matrix from study *i*, which is again assumed fixed and known. The within-study correlation for a given study arises from the use of data from the same set of participants in the estimation of percentile ratios for that study. For random-effects meta-analysis, the vectors of true logPRs are then assumed to be distributed multivariate normally about a vector of combined logPRs **log q**,


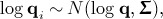
(13)

where ***Σ*** is the between-studies variance–covariance matrix, to be estimated from the data. ***Σ*** comprises the between-studies variances and the between-studies correlation. The between-studies variances measure the variation in the true effect sizes across studies, equivalent to 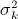 in the aforementioned standard random-effects meta-analysis. The between-studies correlation is the correlation between the true effect sizes across studies.

The model given by (12) and (13) can be estimated using the mvmeta package in stata, which uses restricted maximum likelihood (REML) to estimate **log q** and ***Σ*** 17, 18. In practice, there must be sufficient data available to estimate the *K* variance parameters and the *K*(*K*_− 1_) / 2 correlation parameters contained in the matrix ***Σ***. The number of percentile levels for which estimation is possible is therefore limited by the number of studies in the dataset. The number of parameters to be estimated in ***Σ*** could be reduced by imposing constraints, but options to do this within the mvmeta package are currently limited.

## 3. Simulation study

In this section, we present the results of a simulation study designed to assess the performance of stage 1 of the two-stage method. Bivariate meta-analysis has already been investigated in simulation studies 19. These results can be anticipated to apply to the multivariate meta-analysis of stage 2 of our method; it is not our intention to examine the properties of multivariate meta-analysis here.

Data were simulated from a Cox proportional hazards model with a log-logistic baseline distribution with location parameter *μ* = 4, scale parameter *σ* = 0.3 and log hazard ratio *θ* = − 0.4. The baseline parameter values were chosen to be similar to parameter estimates for the example dataset of Section 4. The hazard ratio was chosen to represent a moderately beneficial effect of treatment. For simplicity, no censoring was assumed to take place, but results should be comparable to censored datasets with a similar numbers of events.

Simulated data were generated for studies with 20, 100 and 500 participants, with equal numbers in each treatment arm, and percentile ratios were calculated for percentile levels 0.9, 0.7, 0.5, 0.3 and 0.1 (we give percentile levels in decreasing orders because higher percentile levels correspond to earlier survival times). Because of the absence of censoring in these simulated datasets, estimated survival probabilities were always equal to the chosen percentile levels for some time interval. Percentile estimates were therefore always calculated using Equation (4). To investigate percentiles estimated using Equation (3), we also generated data for studies with 18, 98 and 498 participants. For each simulated dataset, two variance estimates were calculated, one using the asymptotic expansion of Section 2.1.1 and the other using the bootstrapping method of Section 2.1.2 with 1000 bootstrap samples.

For asymptotic variance estimation, the presmoothing method for estimation of the survival density function requires two bandwidths to be specified, as explained in Appendix B. The ‘presmooth’ package offers two built-in options for bandwidth selection, ‘plug-in’ and ‘bootstrap’. However, we found these to be unreliable for some simulated datasets, giving undersmoothed density function estimates that were inappropriately close to zero. For the simulation study, we instead fixed the bandwidths for all datasets to be the median of the bandwidths selected by the ‘plug-in’ method for 50 simulated datasets for each treatment arm in each study size.

From the simulations study results (not presented here), the bias in the estimated logPRs is estimated to be less than 0.01 for all percentile ratios for all sample sizes except the smallest, *n* = 18 and 20. For the smaller sample sizes, the estimated bias is greatest for the lower percentile levels, rising to 0.086 for *n* = 18 and 0.046 for *n* = 20 at *k* = 0.1. The true logPR at *k* = 0.1 is 0.362.

Results for the estimated coverage probabilities are given in Table I, calculated as the percentage of replications for which the estimated 95% confidence interval (CI) for the logPR contains the true value. The asymptotic variance estimation method appears to perform badly, with only larger sample sizes giving appropriate coverage probabilities of approximately 0.95 for mid-range percentile levels. For higher percentile levels, coverage tends to be too high, whereas for lower percentile levels, it tends to be too low. This is consistent with the first omitted term in the asymptotic expansion (14), which provides a negative contribution for lower percentile levels and a positive contribution for higher levels. In practice, the method could be improved by selecting more appropriate bandwidths, considering each study individually, as in the analysis of the example dataset in Section 4. For the bootstrap method of variance estimation, the estimated coverage is much better, with reasonable coverage for studies with as few as 20 patients for the mid-range percentile levels. For the smaller studies, coverage is too low for the highest percentile levels, where few events have yet to take place, and for low percentile levels, where few patients remain in the study. Computation time is longer for the bootstrap method than for the asymptotic method but is not prohibitively so, with the estimation procedure taking around 1 min for the largest studies considered here.

**Table I:** Estimated coverage of log percentile ratio estimates for data simulated from a log-logistic proportional hazards distribution

		Estimated coverage probability					
		
Variance estimation method	*k*	*n* = 18	*n* = 20	*n* = 98	*n* = 100	*n* = 498	*n* = 500
Asymptotic	0.9	0.992	0.981	0.988	0.986	0.967	0.964
	0.7	0.972	0.966	0.985	0.981	0.968	0.969
	0.5	0.920	0.936	0.963	0.963	0.960	0.957
	0.3	0.800	0.897	0.928	0.930	0.943	0.939
	0.1	0.498	0.892	0.846	0.885	0.925	0.922
Bootstrap	0.9	0.765	0.863	0.960	0.959	0.951	0.952
	0.7	0.962	0.956	0.961	0.952	0.949	0.953
	0.5	0.963	0.954	0.956	0.948	0.955	0.951
	0.3	0.961	0.960	0.954	0.948	0.953	0.948
	0.1	0.744	0.813	0.960	0.957	0.953	0.950

## 4. Example: glioma dataset

We illustrate our method using a dataset consisting of nine studies examining the post-surgery treatment of patients with high-grade glioma. Patients in the treatment groups underwent a post-surgery course of radiotherapy and chemotherapy, whereas in the control groups, patients were treated with radiotherapy alone. The original meta-analysis contained 12 studies. Hazard ratios were estimated by log-rank statistics and combined using fixed-effects meta-analysis, giving a pooled hazard ratio of 1.18 (95% CI 1.09, 1.28) 20. We were unable to obtain data from three of these studies and re-analysed the remaining nine using the two-stage method of Section 2. In our analysis, the studies are labelled 2, 7, 9, 11, 13, 16, 17, 18 and 19, in line with the labels given in the dataset.

The data were investigated for departures from the proportional hazards assumption of Siannis *et al.* 8 by inspecting plots of the estimated log-cumulative hazard against log time (in these plots, the lines for the treatment and control groups should appear parallel under proportional hazards). In these plots, proportional hazards appeared to be violated for trials 9 and 17 and to be questionable for trials 19 and 11.

**Figure 1:**
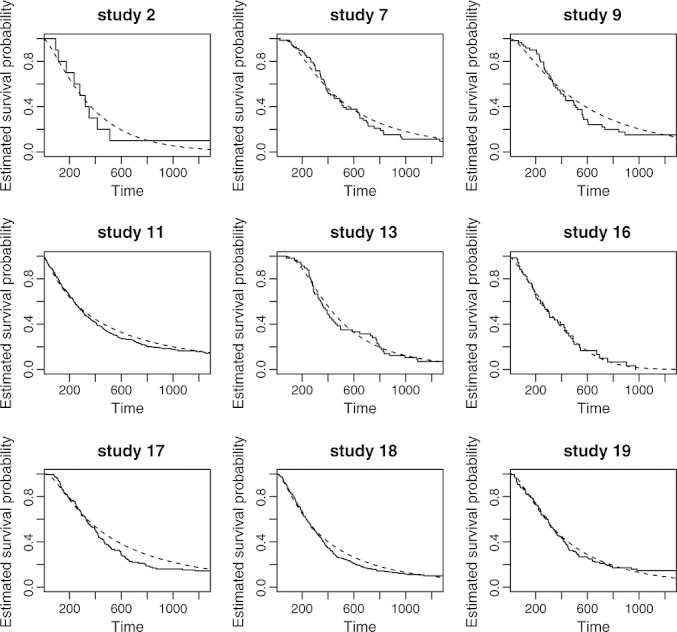
Assessing goodness of fit of the extended-log-gamma distribution to the data from each study. Plotted are estimates of the survival curve for the treatment group with Kaplan–Meier estimates plotted using solid lines and extended-log-gamma estimates using dashed lines.

These data were also analysed using the accelerated failure time models of Siannis *et al.* 8. The distribution used was the extended log-gamma distribution, which incorporates several of the more commonly used accelerated failure time distributions. We have assessed the goodness of fit of the extended-log-gamma distribution to the data from each study by comparing the estimated survival curves from the extended-log-gamma analysis for treatment and control groups with the respective Kaplan–Meier estimates. The plots for the treatment groups are given in Figure 1; plots for control groups are similar. The extended-log-gamma distribution appears to fit the data well for some studies, such as study 16, but not for others, such as studies 9 and 17. The data therefore warrant further investigation using the methods described here.

### 4.1 Implementation

We estimated logPRs for each study in the glioma dataset at *k* = 0.9, …, 0.2 using the two-stage method of Section 2. There were insufficient data available to estimate logPRs at *k* = 0.1. Variance–covariance matrices were calculated for each study using both asymptotic and bootstrap methods.

For the asymptotic variance estimation method, bandwidths were selected using the plug-in option as a default. To ensure that appropriate bandwidths had been selected, estimated density functions were plotted for each treatment arm in each study. For the control arms of studies 9 and 19, the estimated density functions appeared to be undersmoothed. We then used the bootstrap bandwidth selection option as an alternative. If the estimated curves still appeared to be undersmoothed, we then multiplied the bootstrap bandwidth by successive integers until the estimated curves appeared smooth.

When applying the bootstrap variance estimation method, we were unable to calculate the logPR at lower percentile levels for some bootstrap samples when Kaplan–Meier estimates of survival curves did not decrease below a survival probability of 0.2. Removing all bootstrap samples where this is the case may bias the variance estimate because those samples would tend to have higher survival rates in the treatment group and therefore larger differences between treatment groups. For study 9, the logPR at *k* = 0.2 could not be estimated for 140 out of 1000 bootstrap samples and for four out of 1000 at *k* = 0.3. We therefore calculated logPRs only at *k* = 0.9, …, 0.3, discarding the small number of samples at *k* = 0.3 for which logPRs could not be calculated.

The code was written in R for the estimation of percentile ratios and the variance–covariance matrix in stage 1 and is available from the authors on request. In stage 2, multivariate meta-analysis was carried out using the ‘mvmeta’ command in stata with unconstrained between-studies covariance matrix ***Σ***. Parameter values for the multivariate meta-analysis model described in Equations (12) and (13) are estimated using REML 17, 18. Univariate meta-analyses were also carried out using ‘mvmeta’ and REML.

### 4.2 Results

**Figure 2:**
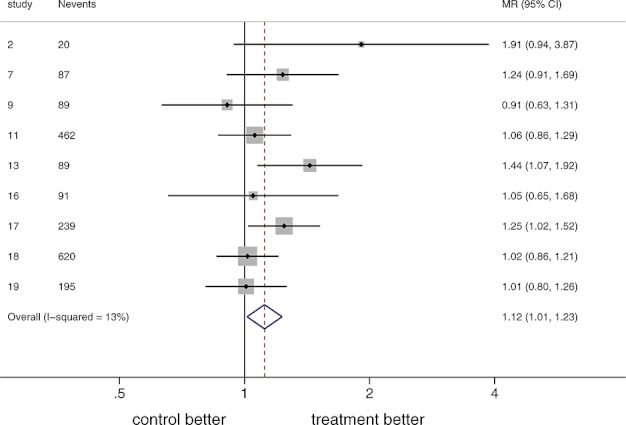
Forest plot of estimated median ratios for the glioma data using bootstrap variance estimation in stage 1 and univariate meta-analysis in stage 2. MR, median ratio, CI, confidence interval.

We first present in Figure 2 a forest plot of median ratios with variances estimated by bootstrapping in stage 1 and combined on the log-scale using univariate, random-effects meta-analysis. The combined median ratio of 1.12 with 95% CI (1.01, 1.23) indicates a small increase in median survival time associated with treatment. This is consistent with a two-stage meta-analysis of the same nine trials using hazard ratios, which gives a combined hazard ratio of 0.85 (95% CI 0.77, 0.93), and with the one-stage analysis of Siannis *et al.* using accelerated failure time models, which gave a combined percentile ratio of 1.18 (95% CI 1.07, 1.29). The widths of the estimated CIs are similar for all analyses, suggesting that the new method has similar power to detect non-null treatment effects compared with the standard method. The value of *I*^2^, which measures the proportion of the variation in the data due to heterogeneity 21, is estimated to be 13%, indicating a low level of heterogeneity in the data.

**Figure 3:**
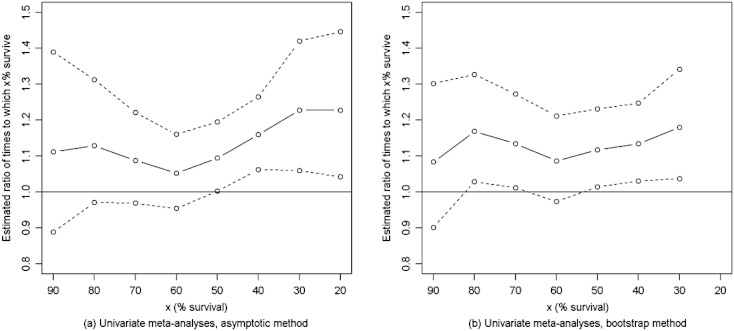
Plots of combined percentile ratio estimates and 95% confidence intervals using (a) asymptotic and (b) bootstrap variance estimation in stage 1 and univariate meta-analysis in stage 2.

We present results for the full range of *k*-values for logPRs combined using univariate meta-analysis in the left-hand side of Table II, illustrated graphically in Figures 3a and 3b. The results using asymptotic variance estimation in stage 1 suggest a slight increase in combined logPR values at later values of *k*, whereas for the bootstrapped results, combined logPRs appear to be more constant over time. Estimated standard errors are also less variable when the bootstrap method is used. Results from the simulation study of Section 3 suggest that the bootstrapped results are more reliable. In general, CIs are wider at early *k*, when fewer events have taken place, and at later *k*, when fewer participants remain in the studies. Also presented in Table II are REML estimates for the between-studies standard deviation 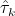. For higher percentile levels, 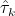 is 0.000, indicating that no or very little heterogeneity is present. Heterogeneity increases as the percentile level decreases, with 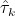 rising to 0.091 for *k* = 3 (using the bootstrap method).

**Table II:** Meta-analysis results for the glioma data with asymptotic and bootstrap variance estimation in stage 1 and univariate and multivariate meta-analysis in stage 2

	Asymptotic univariate		Bootstrap univariate		Asymptotic multivariate		Bootstrap multivariate	
				
*k*	logPR	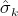	logPR	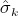	logPR	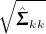	logPR	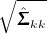
0.9	0.106 (0.114)	0.000	0.080 (0.094)	0.000	—	—	—	—
0.8	0.121 (0.077)	0.000	0.156 (0.065)	0.000	0.095 (0.078)	0.040	0.149 (0.066)	0.051
0.7	0.084 (0.059)	0.000	0.126 (0.059)	0.044	0.074 (0.066)	0.079	0.119 (0.060)	0.067
0.6	0.051 (0.050)	0.000	0.083 (0.056)	0.071	0.048 (0.064)	0.106	0.084 (0.055)	0.078
0.5	0.090 (0.045)	0.000	0.111 (0.049)	0.052	0.091 (0.059)	0.105	0.109 (0.052)	0.082
0.4	0.148 (0.048)	0.032	0.126 (0.049)	0.024	0.150 (0.058)	0.110	0.150 (0.054)	0.089
0.3	0.205 (0.077)	0.160	0.165 (0.066)	0.091	0.197 (0.079)	0.178	0.184 (0.074)	0.132
0.2	0.205 (0.082)	0.151	—	—	—	—	—	—

Reported are parameter estimates with standard errors in brackets and estimates of the between-studies standard deviation (*σ*_*k*_ in the univariate case and 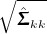 in the multivariate case)

To compare our results with a method based on time-varying hazard ratios, we re-analysed the data using Cox relative risk regression models with different treatment effects in years 1, 2, 3 and beyond of the studies. The combined hazard ratio was 0.84 for year 1 (95% CI 0.72, 0.98), 0.85 for year 2 (95% CI 0.71, 1.02) and 0.86 for year 3 and beyond (95% CI 0.64, 1.15). For this example, both methods are in agreement, suggesting a small, beneficial effect of treatment, which is maintained over time.

Results for logPRs combined using multivariate meta-analysis are presented in the right-hand side of Table II. Only logPRs for *k* = 0.8, …, 0.3 have been combined to avoid convergence difficulties when too many correlation parameters are estimated. The asymptotic and bootstrapped results both show similar trends to those observed in the univariate analyses. The multivariate standard error estimates are slightly higher than the corresponding univariate estimates, in contrast with expectations that the borrowing of strength among outcomes would lead to smaller standard errors. The larger standard errors in the multivariate case appear to be caused by higher estimates of the between-studies standard deviation (also given in Table II). To investigate why this is the case, we consider the estimated between-studies correlation matrices, which are presented in Table III. Recall that the between-studies correlation is the correlation between the true logPRs across studies and is estimated from the data. Riley *et al.* 19 found that estimates of between-studies variance may be inflated in bivariate meta-analysis to compensate for a between-studies correlation estimate of ± 1, which is on the boundary of the parameter space. It is possible that a similar effect may be observed when the estimated correlation is very close to the boundary. In our case, only one of the correlations is estimated to be 1 in Table III, but several correlation parameters are estimated to be very close to 1.

**Table III:** Estimated between-studies correlation matrices from multivariate meta-analysis of the glioma data with asymptotic and bootstrap variance estimation in stage 1

	Asymptotic						Bootstrap					
		
*k*	0.8	0.7	0.6	0.5	0.4	0.3	0.8	0.7	0.6	0.5	0.4	0.3
0.8	1	.	.	.	.	.	1	.	.	.	.	.
0.7	0.772	1	.	.	.	.	0.130	1	.	.	.	.
0.6	0.941	0.941	1	.	.	.	0.196	0.998	1	.	.	.
0.5	0.813	0.998	0.962	1	.	.	− 0.026	0.988	0.975	1	.	.
0.4	0.694	0.993	0.896	0.983	1	.	− 0.257	0.925	0.897	0.973	1	.
0.3	0.676	0.990	0.885	0.979	1.000	1	− 0.335	0.891	0.859	0.951	0.997	1

Our analysis of the glioma dataset suggests that there is limited benefit in the use of multivariate meta-analysis in this context. However, it may still be advantageous to use multivariate meta-analysis for some datasets. For example, if some of the studies had only sufficient follow-up to estimate a subset of the logPRs of interest, multivariate meta-analysis would enable the combination of logPRs that were missing in those studies to borrow strength from the logPRs that had been observed 22. To illustrate this, we re-analysed the glioma data, but with one of the studies artificially truncated by removing estimated logPRs for *k* = 0.5, …, 0.1 for that study. We did this for each study in turn and analysed each truncated dataset using both univariate and multivariate meta-analyses with bootstrapping in stage 1 (we chose to use the bootstrapping method here because it gives better results than the asymptotic method in the simulation results of Section 3). For the multivariate meta-analyses, logPRs at levels *k* = 0.7,0.6,0.5 and 0.4 were combined simultaneously. Results for combined median ratios are presented in Table IV. Recall from Table II that for the full dataset the estimated combined median ratio from the univariate analysis was 0.111 with standard error 0.049. For each truncated study, the multivariate median ratio estimates are closer to the full data estimate than the univariate estimates. This is because data from the truncated studies did not contribute to the univariate meta-analyses because estimated median ratios were not available, whereas the multivariate meta-analyses incorporated information from logPR estimates at *k* = 0.7 and 0.6 for those studies. Estimated standard errors in Table IV are lower in the multivariate meta-analyses for over half the truncated studies. For studies 2, 13 and 17, the univariately estimated standard errors are lower compared with the estimate from the full dataset because the removal of a study has reduced the estimated heterogeneity in the data. In these cases, the multivariate analysis would give a more conservative estimate for the standard error.

**Table IV:** Meta-analysis results for the glioma data with truncated follow-up in one of the studies using bootstrap variance estimation in stage 1 and univariate and multivariate meta-analyses in stage 2

Study	*n*	Analysis	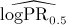 (SE)	*p*-value	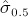
2	20	Univariate	0.101 (0.048)	0.034	0.045
		Multivariate	0.112 (0.051)	0.028	0.073
7	105	Univariate	0.103 (0.053)	0.051	0.060
		Multivariate	0.111 (0.052)	0.032	0.072
9	116	Univariate	0.126 (0.054)	0.019	0.058
		Multivariate	0.122 (0.052)	0.019	0.071
11	511	Univariate	0.126 (0.060)	0.034	0.081
		Multivariate	0.106 (0.055)	0.053	0.084
13	91	Univariate	0.083 (0.045)	0.065	0.000
		Multivariate	0.115 (0.054)	0.033	0.076
16	125	Univariate	0.115 (0.052)	0.028	0.062
		Multivariate	0.116 (0.053)	0.028	0.077
17	270	Univariate	0.082 (0.048)	0.085	0.000
		Multivariate	0.111 (0.054)	0.039	0.071
18	674	Univariate	0.137 (0.055)	0.012	0.054
		Multivariate	0.128 (0.050)	0.010	0.058
19	235	Univariate	0.130 (0.056)	0.021	0.068
		Multivariate	0.121 (0.052)	0.021	0.071

Multivariate meta-analyses combined logPRs simultaneously for *k* = 0.7, 0.6, 0.5, 0.4.

## 5. Discussion

Meta-analyses of survival data usually focus on the estimation of constant hazard ratios. If an assumption of proportional hazards seems inappropriate, then models that incorporate time-varying hazards could be used. However, it may be preferable to consider alternative measures of treatment effect, which are more readily interpretable. We have proposed a novel, two-stage method for meta-analysis of survival IPD using percentile ratios. The advantage of our method lies in the avoidance of distributional assumptions at the study level, which makes it suitable to use when proportional hazards or accelerated failure time assumptions are inappropriate.

Stage 1 of the proposed meta-analysis involves estimation of logPRs and their variance–covariance matrix from Kaplan–Meier estimates of the survivor function. We have presented two methods for estimation of the variance–covariance matrix, an asymptotic method and a bootstrap method. Results from the simulation study of Section 3 suggest that the bootstrap method has the superior coverage properties, and we therefore recommend its use for variance estimation. If bootstrapping is too computationally intensive, the asymptotic method could be used to estimate mid-range logPRs for sufficiently large studies.

In stage 2, logPRs can be combined using either univariate or multivariate meta-analysis. When logPR estimates are available for all percentile levels of interest, we recommend the use of univariate meta-analysis because multivariate meta-analysis can lead to inflated estimates of between-studies variances. However, when some studies lack sufficient data to estimate all logPRs, then a multivariate meta-analysis may be more appropriate to allow combined logPR estimates to borrow strength across percentile levels. We have not yet explored the possibility of constraining the between-studies variance–covariance matrix ***Σ*** to have, for example, an auto-correlated form; we leave this for future work.

Our method could be adapted to allow either parametric or non-parametric methods to be used in stage 1 of a two-stage meta-analysis. Where appropriate, study-level data could be modelled using parametric distributions, with the non-parametric approach being used when distributional assumptions appear unjustified. Alternatively, the non-parametric method could be extended to estimate other quantities of interest from survival curves, such as percentile differences, ratios of survival probabilities at a given time, or differences in survival probabilities. Further work would be required to investigate the asymptotic properties of these alternative measures of treatment effect.

Our focus has been on methods for meta-analysis of IPD. Our approach could also be used in the meta-analysis of aggregate data if methods were available for the extraction of IPD from published survival curves. To reconstruct the individual patient data, a model would be required for the censoring mechanism. Parmar *et al.* 23, Williamson *et al.* 24, Ouwens *et al.* 7 and Fiocco *et al.* 25 have recently made progress in this area.

## Appendix A. Derivation of variance–covariance formulae

### A.1 Derivation of variance formula

We derive the formula (5), following the method described in 26, by asymptotic expansion of the survival function

(14)

Rearranging and using *f*(*t*) = − d*S* / d*t*, we get

(15)

By the delta method

(16)

and using equation (15) we obtain

(17)

### A.2 Derivation of covariance formula

Let *t*_(1)_, …, *t*_(*N*)_ be the *N* observed event times for the study in question and let  be the estimated probability of surviving through the time interval that begins at *t*_(*m*)_. Then the estimated survivor function at time *t* is given by

(18)

where *t*_(*r*)_ *≤ t* < *t*_(*r* + 1)_. Suppose *t*_1_ *≤ t*_2_, then

(19)

where we have used the assumption that 's corresponding to different time intervals are independent. Then, by using asymptotic expansions of  in both sides of (19) we obtain

(20)

Then, using similar calculations to those described in section A.1, and approximating  by (20), we obtain

(21)

## Appendix B. Presmoothed density function estimation

A presmoothed kernel density estimator for censored data was introduced by Cao and Jacome in 27. See also 28 for an extension to left-truncated data.

Let *Z*_*i*_ be the ordered observation times, *i* = 1, …, *n*, with *Z*_*i*_ = min(*T*_*i*_,*C*_*i*_), where *T*_*i*_ is the event time and *C*_*i*_ is the censoring time. Let *δ*_*i*_ be the censoring indicator with *δ*_*i*_ = 1 if the observation is uncensored and *δ*_*i*_ = 0 otherwise. Then the Kaplan-Meier estimate  of the probability distribution function *F*(*t*) of T is given by

(22)

An estimate  for the probability density function *f*(*t*) can be derived from  using the standard kernel density estimation procedure due to Rosenblatt 29 and Parzen 30:

(23)

where *K*(*x*) is a kernel and *K*_*s*_(*x*) = *s*^− 1^*K*(*x* / *s*) is the rescaled kernel with bandwidth *s*.

Let *m*(*t*) be the conditional probability of uncensoring, *m*(*t*) = *E*(*δ* | *Z* = *t*). The function *m*(*t*) can be estimated non-parametrically using the Nadaraya–Watson estimator with kernel *K* and bandwidth *b*

(24)

Replacing the censoring indicator *δ*_*i*_ in (22) with  leads to the presmoothed estimate  of *F*(*t*):

(25)

Note that the presmoothed estimate  is a step-like function of *t* with jumps at all values of *Z*_*i*_. It has been shown to be more efficient than the Kaplan-Meier version  for a suitable choice of the bandwidth *b*. Analogous to equation (23), a corresponding presmoothed estimate  of *f*(*t*) is then

(26)

Note that the estimate  depends on two bandwidths; the presmoothing bandwidth *b*, used in the estimation of *m*(*t*), and the smoothing parameter *s*, used to estimate *f*(*t*) from .

Efficiency of the presmoothed estimator  relies on selection of appropriate bandwidths *b* and *s*. In 31 two approaches to bandwidth selection are described, both of which have been implemented in the presmooth package. The first approach, termed ’plug-in’ bandwidth selection, minimises an asymptotic expansion of the mean integrated squared error (MISE). The second approach, ’bootstrap’ bandwidth selection, minimises a bootstrap version of the MISE. See 31 for details.

## References

[1] Simmonds MC, Higgins JP, Stewart LA, Tierney JF, Clarke MJ, Thompson SG (2005). Meta-analysis of individual patient data from randomized trials: a review of methods used in practice. Clinical Trials.

[2] Group EBCTC (1988). Effects of adjuvant tamoxifen and of cytotoxic therapy on mortality in early breast cancer. The New England Journal of Medicine.

[3] Moodie PF, Nelson NA, Koch GG (2004). A non-parametric procedure for evaluating treatment effect in the meta-analysis of survival data. Statistics in Medicine.

[4] Arends LR, Hunink MGM, Stijnen T (2008). Meta-analysis of summary survival curve data. Statistics in Medicine.

[5] Dear KBG (1994). Iterative generalized least squares for meta-analysis of survival data at multiple times. Biometrics.

[6] Fiocco M, Putter H, van Houwelingen JC (2009). Meta-analysis of pairs of survival curves under heterogeneity: A Poisson correlated gamma-frailty approach. Statistics in Medicine.

[7] Ouwens MJNM, Philips Z, Jansen JP (2010). Network meta-analysis of parametric survival curves. Research Synthesis Methods.

[8] Siannis F, Barrett JK, Farewell VT, Tierney JF (2010). One-stage parametric meta-analysis of time-to-event outcomes. Statistics in Medicine.

[9] Jackson D, Riley R, White IR (2011). Multivariate meta-analysis: Potential and promise. Statistics in Medicine.

[10] Sander JM (1975). The weak convergence of quantiles of the product-limit estimator. Technical Report 5.

[11] Roth AJ (1985). Variances of the kaplan-meier estimator and its quantiles under certain fixed censoring models. The Annals of Statistics.

[12] Reid N (1981). Influence functions for censored data. The Annals of Statistics.

[13] Lopez-de Ullibarri I, Jacome MA (2011). survPresmooth: Presmoothed estimation in survival analysis. http://CRAN.R-project.org/package=survPresmooth,rpackageversion1.1-1.

[14] Efron B (1981). Censored data and the bootstrap. Journal of the American Statistical Association.

[15] Efron B, Gong G (1983). A leisurely look at the bootstrap, the jackknife and cross-validation. The American Statistician.

[16] Riley RD (2009). Multivariate meta-analysis: the effect of ignoring within-study correlation. Journal of the Royal Statistical Society: Series A (Statistics in Society).

[17] White IR (2009). Multivariate random-effects meta-analysis. The Stata Journal.

[18] White IR (2011). Multivariate random-effects meta-regression: updates to mvmeta. The Stata Journal.

[19] Riley R, Abrams K, Sutton A, Lambert P, Thompson J (2007). Bivariate random-effects meta-analysis and the estimation of between-study correlation. BMC Medical Research Methodology.

[20] Meta-analysis Trialists Group G (2002). Chemotherapy in adult high-grade glioma: a systematic review and meta-analysis of individual patient data from 12 randomised trials. The Lancet.

[21] Higgins JPT, Thompson SG (2002). Quantifying heterogeneity in a meta-analysis. Statistics in Medicine.

[22] Collaboration FS (2009). Systematically missing confounders in individual participant data meta-analysis of observational cohort studies. Statistics in Medicine.

[23] Parmar MKB, Torri V, Stewart L (1998). Extracting summary statistics to perform meta-analyses of the published literature for survival endpoints. Statistics in Medicine.

[24] Williamson PR, Smith CT, Hutton JL, Marson AG (2002). Aggregate data meta-analysis with time-to-event outcomes. Statistics in Medicine.

[25] Fiocco M, Stijnen T, Putter H (2012). Meta-analysis of time-to-event outcomes using a hazard-based approach: Comparison with other models, robustness and meta-regression. Computational Statistics and Data Analysis.

[26] Collett D (2003). Modelling Survival Data in Medical Research, Second Edition.

[27] Cao R, JÃącome M (2004). Presmoothed kernel density estimator for censored data. Journal of Nonparametric Statistics.

[28] Jacome MA, Iglesias-Perez MC (2010). Presmoothed estimation of the density function with truncated and censored data. Statistics.

[29] Rosenblatt M (1956). Remarks on some nonparametric estimates of a density function. The Annals of Mathematical Statistics.

[30] Parzen E (1962). On the estimation of a probability density function and mode. Annals of Mathematical Statistics.

[31] Jacome MA, Cao R (2008). Asymptotic-based bandwidth selection for the presmoothed density estimator with censored data. Journal of Nonparametric Statistics.

